# A review of hypoglycaemia in a South African family practice setting

**DOI:** 10.4102/phcfm.v8i1.1095

**Published:** 2016-06-17

**Authors:** Devan K. Pillay, Andrew J. Ross, Laura Campbell

**Affiliations:** 1 Department of Family Medicine, University of KwaZulu-Natal, South Africa; 2 Family Medicine, School of Nursing and Public Health, University of KwaZulu-Natal, South Africa

## Abstract

**Background:**

The prevalence and incidence of diabetes in South Africa are high and are expected to increase. Mortality and morbidity may be related to hypoglycaemia, and there is limited information on hypoglycaemia from private practice sites. The aim of this study was to assess patients’ education about, knowledge of and response to hypoglycaemia.

**Methods:**

The study site was a general practice, and participants were all patients with diabetes who presented to the practice over a 1-month period. Data were collected using a closed-ended questionnaire and analysed descriptively.

**Findings:**

Most respondents were South Africans of Indian origin and were diagnosed with diabetes at a relatively young age. Despite attending a private practice, most had low incomes and low schooling levels. Just under half reported having experienced hypoglycaemia, and there was a strong association between hypoglycaemia and insulin use. Many reported never having received any education around hypoglycaemia.

**Discussion:**

The study highlights the need for early screening for diabetes in this vulnerable population. Hypoglycaemic education should consider low schooling levels even in a private general practice, and further study is required on the quality and frequency of education provided in general practice.

## Background

Throughout sub-Saharan Africa the incidence and prevalence of diabetes mellitus (DM) remain high, and the general population-prevalence is expected to increase from 4.3% to 5.0% between 2012 and 2030.^1^ The rise in prevalence and associated diabetic morbidity and mortality is multifactorial and includes rapid urbanisation, obesity, physical inactivity, low income and its influence on social habits, lack of healthcare infrastructures, lack of access to healthcare professionals and patients’ poor knowledge of the disease.^1^ Several of these factors such as urbanisation, obesity and lack of access to healthcare professionals are pertinent in the South African context. In South Africa (SA), the overall prevalence of DM is estimated to be 5.5% for people older than 30 years of age.^2^ However, DM is known to be particularly common among South Africans of Indian origin,^3^ among whom the prevalence is estimated to be as high as 17.1% in comparison to 6.4% among urbanised African people and 6.2% among White and Coloured people.^2^

Iatrogenic hypoglycaemia is an acute complication in both type 1 and type 2 DM and is because of the use of oral sulphonylureas and insulin in the management of DM. Hypoglycaemia occurs more frequently in insulin-deficient states (type 1) and in advanced insulin-resistant states (severe type 2).^4^ Severe hypoglycaemia in any type of diabetes is associated with increased risks of morbidity and mortality, especially in patients with comorbid cardiovascular risks, and in the elderly.^4^ Although direct causality was not demonstrated, a strong association between severe hypoglycaemia and an increased risk of death was shown in the Action to Control Cardiovascular Risk in Diabetes study.^5^ Moreover, quality of life decreases as the frequency of hypoglycaemic episodes increases.^6^ Consequently, prevention and control of hypoglycaemia is one of the major considerations for the primary care physicians when adopting a patient-centred approach to the management of DM.^7^

Most reports on the incidence of hypoglycaemia arise from clinical trials such as the Diabetes Control and Complications Trial and the United Kingdom Prospective Diabetes Study.^8,9^ These incidence studies were carried out in developed countries. Literature notes that it is difficult to accurately assess the frequency of hypoglycaemia in real life, and most estimates of frequency have been gleaned from studies based on retrospective data from registries or databases.^10^ There is less literature on the incidence of hypoglycaemia in developing countries such as SA and even less data on the experiences of hypoglycaemia among patients attending a private general practice. In addition, there is limited literature on patient-related factors which influence hypoglycaemia such as patients’ knowledge of the disease and of hypoglycaemia.

Literature reports that it is vital for both patients and caregiver to receive education around hypoglycaemia to ensure recognition of warning signs and appropriate acute intervention in a home or work situation.^11^ Education is required around whether the patient should be admitted immediately to a hospital or whether the primary care physician can be contacted to offer advice or visit at home.

This study considered knowledge of the symptoms of hypoglycaemia among patients attending a private general practice and is important because it was carried out in a developing world context, employed real life data from patients and honed in on patients’ reported experiences of and education around hypoglycaemia. The study may firstly guide primary care physicians in planning for the care of patients with diabetes (PWD) and their caregivers. Secondly the study may alert researchers to important topics for further investigation. The study questions were threefold: (1) what are the demographic profiles of patients who have diabetes in a private general practice; (2) what are the hypoglycaemic experiences of these patients; and (3) what hypoglycaemic education have patients reported to have received?

## Methods

The study design was exploratory, descriptive and prospective. The aim of this study was to assess patients’ knowledge of the symptoms of hypoglycaemia, how they responded to episodes of hypoglycaemia and what information they had received about hypoglycaemia. The intention was to better understand this particular practice population and guide further research even if the results are not necessarily generalisable to a larger South African diabetic population.

The study site was a busy urban private general practice in KwaZulu-Natal, South Africa. The practice is situated in an area where the population is mainly South Africans of Indian origin. The practice sees a mixture of medical aid and cash-paying patients (± 30% and ± 70%, respectively). The practice staff includes one doctor, one registered nurse and one diabetic educator. Medical aids pay for diabetic patients to receive intensive diabetic education and to see a dietician, podiatrist and ophthalmologist annually. These services are available to all patients based on their ability to pay for the services. The researcher, an accredited diabetic care provider who has a keen interest in diabetes care, is based in the practice and has completed a course in diabetes management from the Centre for Diabetes and Endocrinology.

All PWD who attended the study site during the month 1/6/2015 – 30/6/2015 were potential study participants. The population of patients suffering from diabetes that attended this family practice was estimated at 1500, with 250 of these patients attending for diabetes review monthly.

During the mentioned study period all PWD over the age of 18 years who presented at the practice were approached systematically by the nurse on arrival and invited to partake in the study. Participant files were marked to ensure that no one completed the questionnaire on more than one occasion. Information on the study was provided by either the researcher or a practice nurse trained in the study by the researcher.

Data were collected using a questionnaire which was developed around the aims of the study and comprised of the following three sections: (1) demographic details, (2) reported hypoglycaemic episodes and (3) reported education. Demographic details included race (considered important because of the high prevalence of diabetes among South Africans of Indian origin), age at onset of diabetes, education level and income level. The section on ‘Hypoglycaemic episodes’ reviewed type of medication, number of episodes and persons present during an episode. The section on education asked about information the patient received about symptoms and management of hypoglycaemia, who gave the information, and patients were asked to rate the quality of the information given. The questions were derived from literature pertaining to hypoglycaemia and were largely presented as open-ended questions. Content understanding was validated by a pilot study of 10 PWD to check their understanding of the questions and their responses. The questionnaire was modified following this pilot study. Data from the pilot study were excluded from the final analysis. Due to the variation in the literacy level of the practice population the practice nurse was available to assist the PWD during completion of the questionnaire to clarify any queries and correct any misunderstandings.

Data from the completed questionnaires were entered in the SPSS (version 23.0) programme. Descriptive analysis reported means, ranges and standard deviations. Inferential techniques to assess associations between various variables included the use of chi-square test values which are interpreted using *p*-values with a value of < 0.05 considered to be significant.

Permission to conduct this study was obtained from the Biomedical Research Ethics Committee at the University of KwaZulu-Natal (BE 485/14) and patients signed informed consent forms before completing the questionnaire.

## Findings

During the study period (1st – 30th June 2015), 249 PWD attended the study site and all consented to complete the questionnaire. Findings are presented in the following three sections: (1) biographical profile; (2) reported hypoglycaemic profile; and (3) reported hypoglycaemic education.

### Biographical profile

The vast majority of respondents described themselves as South Africans of Indian origin (98%). The remainder were coloured people (4; 0.01%) and there were no people or white people. The average age at diagnosis of DM was 45 – 46 years (mean 46.8 years) and a *p*-value of 0.786 indicated no significant differences between race and age at diagnosis.

The mean duration of treatment for South Africans of Indian origin was 9.9 years (range 3 months – 37 years), while the mean duration of treatment for coloured patients was 23 years (range 2 – 40 years).

**FIGURE 1:**
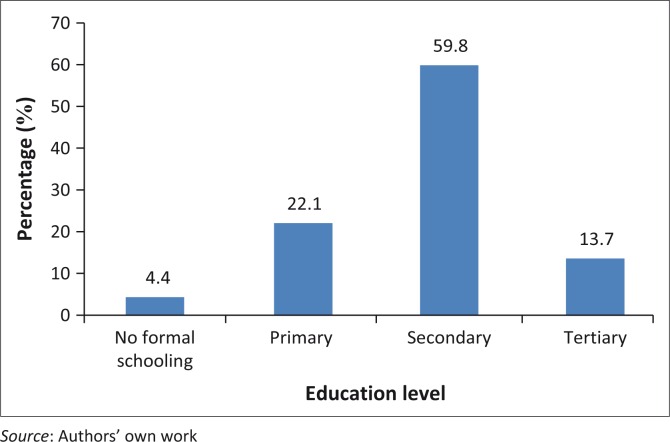
Educational level

A minority (11; 4.4%) had no formal schooling and few (34; 13.7%) had a tertiary education. There was a significant association between age of diagnosis and schooling level with patients who had a tertiary education being diagnosed at an earlier age than those without and conversely, those with no formal education were diagnosed at relatively older ages (*p* = 0.003). The education levels of respondents are summarised in Figure 1.

A quarter of respondents (63; 25.3%) earned more than R10 000 per month and less than 1% (2; 0.8%) were unemployed. Just under half (104; 42%) earned less than R2000 per month. Patients with relatively lower incomes (less than R2000 per month) were diagnosed at a later age than those with higher monthly incomes, (*p* = 0.018).

**FIGURE 2:**
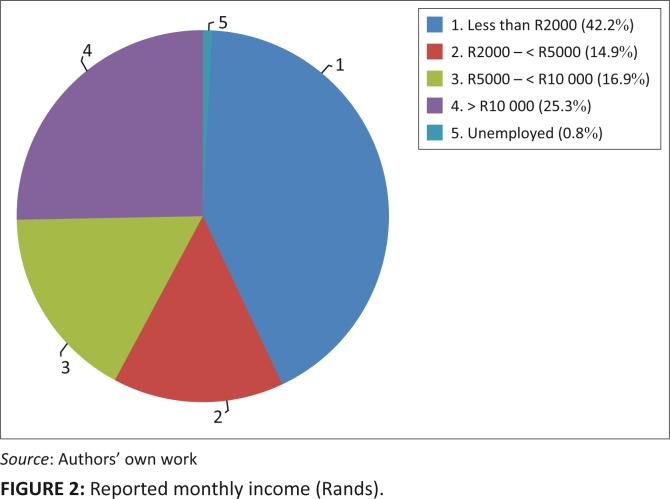
Reported monthly income (Rands).

The reported income per month is summarised in Figure 2.

### Reported hypoglycaemic profile

Most respondents were not using insulin (167; 67%) with just under a third (76; 31%) reporting use of insulin. Of those taking insulin, most self-administered it (68; 88%) at intervals of once (15; 22%), twice (41; 60%) or thrice (12; 17%) per day. A minority relied on a caregiver to administer insulin (8; 10%) and the insulin was delivered once a day (2; 2.5%), twice a day (4; 5%) and thrice a day (2; 2.5%). One patient reported that they went to hospital twice per day for insulin administration (1; 1%).

Just over half reported never having experienced a hypoglycaemic episode (138; 56.6%). The relationship between treatment type (insulin or no insulin) and hypoglycaemia is represented in the Table 1.

**TABLE 1:** Treatment type and reported hypoglycaemia.

Using insulin	Experienced hypoglycaemia		Total *n*(%)

	Yes *n*(%)	No *n*(%)	
Yes	56 (23.0)	21 (8.6)	77.0 (31.6)
No	50 (20.5)	117 (48.0)	167 (68.4)

**Total**	**106 (43.4)**	**138 (56.6)**	**244 (100.0)**

*Source*: Authors’ own work

*n*= count.

There was a strong association between taking insulin and reporting a hypoglycaemic episode (*p* = 0.05). The odds of a patient who was using insulin reporting hypoglycaemia was six times that of a patient not taking insulin. Of the 167 patients not using insulin, 77 were on metformin only and 90 were on metformin and sulphonylureas. Patients on sulphonylureas were 2.63 times more likely to experience hypoglycaemia than patients on metformin only (35/90 v’s 15/77).

**FIGURE 3:**
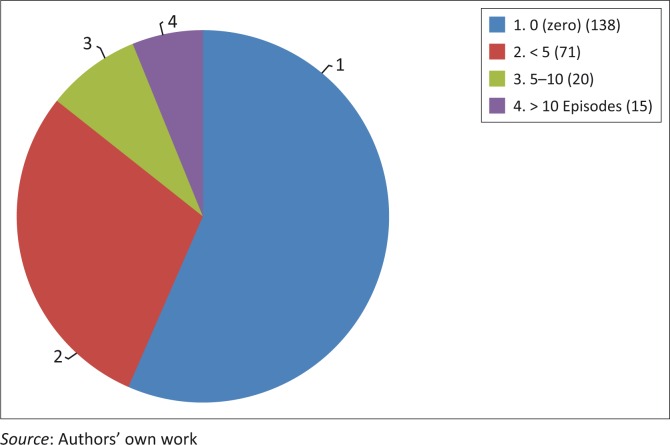
Number of episodes of hypoglycaemia since diagnosis of diabetes.

Over a hundred respondents (106; 42%) reported a hypoglycaemic episode. Seventy one of these respondents (67%) reported that they had experienced less than five episodes since diagnosis and thirty five (33%) had experienced more than five. The reported number of episodes of hypoglycaemia since diagnosis is illustrated in Figure 3.

Most reported that there was someone with them when they experienced a hypoglycaemic episode (68: 66%) and 37 were alone during at least one of the episodes (34%).

Of those who reported hypoglycaemia, most (60; 56%) reported that they recognised the symptoms of hypoglycaemia and took steps to rectify the low sugar levels. The remaining respondents (46:44%) who reported hypoglycaemia relied on others for assistance. Most recovered from the episode in their own homes (92; 87%), some (12; 11%) were taken to hospital and two (2%) recovered at work. There was a non-statistical association between a higher frequency of reported hypoglycaemic episodes (> 5 since diagnosis) (*p* = 0.92) and lower levels of schooling. There was a non-statistical association between more reported episodes of hypoglycaemia in those who earned less than R2000 per month and those who earned more than R2000 per month (*p* = 0.34). There was a significant association between the age of onset of diagnosis and the risk of reporting hypoglycaemia (*p* = 0.005) with those in the age group 40 to 49 years at diagnosis representing the highest prevalence (16 people reporting more than five episodes).

There was no association between the number of times per day which insulin was administered and reported episodes of hypoglycaemia. Similarly, there was no association between self-administered insulin and insulin administered by caregiver and reported hypoglycaemic episodes.

### Reported hypoglycaemic education

A third (96; 39%) reported that they had received no education on diabetes/hypoglycaemia. A chi-square test revealed that there was a significant association between a lower level of schooling and a report of not being educated about diabetes (*p* = 0.001). Of those who reported that they had received education (148; 61%), most were educated/counselled by a doctor (105; 71%), 18 by a nurse educator (12%) and 25 (17%) reported that a diabetic educator informed them about the symptoms and management of hypoglycaemia.

Respondents were asked to comment on their views of the hypoglycaemic education and most (124; 84%) reported the education as good. The remainder (23; 16%) reported that education was average or poor. Of those who rated their education as good, 21 experienced more than five episodes of hypoglycaemia (14 %). The relationship between hypoglycaemic education and frequency of hypoglycaemic episodes is illustrated in Table 2.

**TABLE 2:** Hypoglycaemic education and reported hypoglycaemia.

Episodes of hypoglycaemia	Rate quality of education received					Total

	Missing	Good	Average	Poor	No education	
< 5	1	103	18	1	86	209
≥ 5	0	21	3	1	10	35

**Total**	**1**	**124**	**21**	**2**	**96**	**244**

*Source*: Authors’ own work

The odds of those who were not educated about hypoglycaemic experiencing hypoglycaemia were four times greater than those who received hypoglycaemic education. Conversely, there was a significant relationship between being educated about hypoglycaemia and the number of hypoglycaemic episodes with just under half of the respondents (57; 45%) who were educated around hypoglycaemia reporting < 5 episodes (*p* = 0.027).

## Discussion

This exploratory study primarily aimed to guide a primary care physician’s practice around diabetic care and honed in on the important topic of hypoglycaemia as this could be used as a surrogate marker of diabetic control at both an individual–patient level and at a broader societal level. Secondly, the study aimed to provide direction for future research. The sample consisted mostly of South Africans of Indian origin and there are key lessons to be learnt from this vulnerable population. The profile of patients choosing to use a private general practitioner is not unusual with a large number of low income patients, not on medical aid, choosing to attend a GP practice where they are known to the doctor and ensured of continuity of care rather than accessing free treatment at government facilities. This has implications for the kind of services which this group of patients can afford with some patients not being able to afford visits to the podiatrist or ophthalmologist, relying instead on services provided by the general practitioner.

Most respondents were first diagnosed in their early 40s. Literature highlights that more than half of the people with diabetes are completely unaware of their condition. This study supports a need for screening for diabetes beginning at least in the late 30s or early 40s.^12^ Despite the study being conducted at a private practice, the majority of respondents had a low monthly income, with literature reporting that low income is a major obstacle to the prevention, early detection and control of diabetes.^12^ In low income situations, there can be poor access to healthcare services and thus the involvement of a primary care physician becomes essential.

The schooling levels of respondents varied from no formal schooling to a tertiary education and those who had low schooling levels reported less education around hypoglycaemia. This association between a lack of reported hypoglycaemic education and lower schooling requires further investigation as the respondents may have received some hypoglycaemic education but perhaps this was not in a format that they understood or found to be of use. This study did not specifically review the type of hypoglycaemic education received by respondents and studies elsewhere indicate that most diabetic-patient education relies heavily on written material about disease processes, medical management and self-care instructions.^13^ Despite the availability of extensive written health education materials with relatively consistent content, many materials are too complex to be understood by patients with low literacy levels.^13^ Thus, patients with inadequate literacy may not benefit from educational efforts. In the study population, information around screening, prevention and treatment should be available for all levels of patient literacy and may be useful if presented in non-written ways, for example, videos.

The use of insulin was associated with a six times higher risk of hypoglycaemia. However, it is worth noting that hypoglycaemia was also experienced by respondents on sulphonylureas. Literature supports this finding in that any intensive treatment for diabetes including oral treatment is associated with an increased risk of hypoglycaemic episodes.^14^ The United Kingdom Prospective Diabetes Study reported that 2.5% PWD aged 25 to 65 years reported substantive hypoglycaemic events (grades 2 to 4) each year.^15^ This highlights the importance of ongoing disease monitoring and hypoglycaemic education for all PWD whether a patient is taking insulin or not.

Many of the respondents reported that they were alone during a hypoglycaemic episode and this has serious implications for any hypoglycaemic education programme in that patients must be warned that they may lose consciousness during an episode: in a study of a large cohort of PWD in India in 2011, a quarter of respondents reported that they lost consciousness due to hypoglycaemia.^16^ PWD, their families, friends and co-workers require education around the risks and management of hypoglycaemia and all PWD should be encouraged to obtain medical alert bracelets so that if found unconscious a diagnosis of hypoglycaemia could be rapidly confirmed. The vital role of educating ‘significant others’ in reducing hypoglycaemic morbidity and mortality is stressed in literature.^17^

Effective diabetic education has long been acknowledged to be essential in the prevention and management of hypoglycaemia, and education is widely accepted as the cornerstone of successful diabetes management. Most respondents had received some education around hypoglycaemia from a doctor with fewer reporting having received education from a nurse or diabetic educator. It is not known whether the hypoglycaemic education was as a once-off event or took place more frequently. The topics of quality and frequency of education about hypoglycaemia require further study.

Studies describe that hypoglycaemic education is most successful when it expands beyond the PWD and their families into the broader society.^18^ Community participation and integration of the government and private sectors into any diabetic education endeavour is necessary^18^ and the primary care physician could act in a coordinating role. Annual auditing of patients’ understanding of diabetes and of hypoglycaemic education are complementary to a successful diabetic education programme^19^ and auditing of hypoglycaemic education in this study context may form a useful area for further study.

## Limitations to the study

The study was carried out in a private practice serving a population predominately of South Africans of Indian origin and the findings may not be generalisable to other PWD in SA. Although the study aimed specifically at gaining real life data from PWD, a major limitation is that the hypoglycaemic episodes were based on the patient’s subjective experience of symptoms and not on capillary glucose levels. As reported hypoglycaemic episodes were not verified there may have been a tendency to under or over report episodes of hypoglycaemia. Whether PWD received hypoglycaemic education was also ascertained subjectively and patients may have received education but not remembered this. The quality and frequency of hypoglycaemic education were not considered and this important area requires further study.

## Conclusion

This study has significant implications for primary care physicians caring for PWD. Firstly, it highlights a need for diabetic screening among South Africans of Indian origin beginning at a relatively early age. Secondly, it illustrates that PWDs’ literacy level, even in a private general practice, may be low and this should be taken into consideration when planning any hypoglycaemic education programme. Thirdly, it reminds practitioners that hypoglycaemia can occur in PWD on insulin and those on oral medication. Those on insulin are much more susceptible and hypoglycaemic education may need to be intensive and ongoing. Healthcare providers responsible for caring for PWD need to develop robust systems, such as developing a checklist for giving information about hypoglycaemia to all PWD which must be signed off by the primary care physician.

In addition, subjective data around reported episodes of hypoglycaemia could be triangulated by associating reported episodes with clinical data. The quality, frequency and PWDs’ understanding of hypoglycaemic education form a vital area for future research. Expanding education beyond PWD and their families into the community may also form a useful topic for future investigation.
